# *Veronica* Plants—Drifting from Farm to Traditional Healing, Food Application, and Phytopharmacology

**DOI:** 10.3390/molecules24132454

**Published:** 2019-07-04

**Authors:** Bahare Salehi, Mangalpady Shivaprasad Shetty, Nanjangud V. Anil Kumar, Jelena Živković, Daniela Calina, Anca Oana Docea, Simin Emamzadeh-Yazdi, Ceyda Sibel Kılıç, Tamar Goloshvili, Silvana Nicola, Giuseppe Pignata, Farukh Sharopov, María del Mar Contreras, William C. Cho, Natália Martins, Javad Sharifi-Rad

**Affiliations:** 1 Student Research Committee, School of Medicine, Bam University of Medical Sciences, Bam 44340847, Iran; 2 Department of Chemistry, NMAM Institute of Technology, Karkala 574110, India; 3 Department of Chemistry, Manipal Institute of Technology, Manipal Academy of Higher Education, Manipal 576104, India; 4 Institute for Medicinal Plants Research “Dr. Josif Pančić”, Tadeuša Košćuška 1, Belgrade 11000, Serbia; 5 Department of Clinical Pharmacy, University of Medicine and Pharmacy of Craiova, Craiova 200349, Romania; 6 Department of Toxicology, University of Medicine and Pharmacy of Craiova, Craiova 200349, Romania; 7 Department of Plant and Soil Sciences, University of Pretoria, Gauteng 0002, South Africa; 8 Department of Pharmaceutical Botany, Faculty of Pharmacy, Ankara University, Ankara 06100, Turkey; 9 Department of Plant Physiology and Genetic Resources, Institute of Botany, Ilia State University, Tbilisi 0162, Georgia; 10 Department of Agricultural, Forest and Food Sciences, University of Turin, I-10095 Grugliasco, Italy; 11 Department of Pharmaceutical Technology, Avicenna Tajik State Medical University, Rudaki 139, Dushanbe 734003, Tajikistan; 12 Department of Chemical, Environmental and Materials Engineering, University of Jaén, 23071 Jaén, Spain; 13 Department of Clinical Oncology, Queen Elizabeth Hospital, Hong Kong SAR 999077, China; 14 Faculty of Medicine, University of Porto, Alameda Prof. Hernâni Monteiro, 4200-319 Porto, Portugal; 15 Institute for Research and Innovation in Health (i3S), University of Porto–Portugal, 4200-135 Porto, Portugal; 16 Zabol Medicinal Plants Research Center, Zabol University of Medical Sciences, Zabol 61615-585, Iran

**Keywords:** *Veronica* plants, speedwell, iridoids, phenolic compounds, natural preservatives

## Abstract

The *Veronica* genus, with more than 200 species, belongs to the Plantaginaceae family and is distributed over most of the Northern Hemisphere and in many parts of Southern Hemisphere. These plants are traditionally used in medicine for wound healing, in the treatment of rheumatism, and in different human diseases. This paper reviews the chemical composition of some valuable *Veronica* species, the possibilities *Veronica* extracts have in food preservation and as food ingredients, and their functional properties. *Veronica* species represent a valuable source of biological active secondary metabolites, including iridoid glycosides and phenolic compounds. In particular, due to presence of these phytochemicals, *Veronica* species exhibit a wide spectrum of biological activities, including antimicrobial and antioxidant. In fact, some studies suggest that some *Veronica* extracts can inhibit foodborne pathogens, such as *Listeria monocytogenes*, but only a few of them were performed in food systems. Moreover, anticancer, anti-inflammatory, and other bioactivities were reported in vitro and in vivo. The bioactivity of *Veronica* plants was demonstrated, but further studies in food systems and in humans are required.

## 1. Introduction

The genus *Veronica* at present belongs to the family Plantaginaceae, while it was previously classified in the family Scrophulariaceae [1]. There are many suggestions (problems) related to the classification and rearrangement of this genus [2,3]. The family includes 120 plant genera with 7055 scientific plant names, of which 1614 are accepted species names [4]. The Plant List includes 1520 scientific plant names for the *Veronica* genus. Of these, 234 are accepted species names, 335 are synonyms, and 951 are unassessed. Thus, the total number of species belonging to the genus *Veronica* depends on synonym acceptance. These species are distributed over the Northern Hemisphere and into the Australasian region (Australia, New Zealand, New Guinea), with centers of diversity in western Asia and New Zealand [2]. Most of the species of *Veronica* occur in regions with a Mediterranean precipitation regime from the sea to high alpine elevations. Despite the importance in many habitats, aquatic plants of *Veronica* are mostly researched in modern biosystematic studies. The common member of the semi-aquatic plants in the Mediterranean region is *Veronica* sect. *beccabunga* [5]. Some other common species of *Veronica* genus are represented in Table 1.

Traditional medicine is a sum of great knowledge about health, disease prevention, treatment, and physical and mental illnesses. There are different types of traditional medicine such as ancient Iranian medicine, traditional Chinese medicine, Ayurveda, traditional African medicine, acupuncture, and so on [7]. In 2019, the World Health Organization published a global report on traditional and complementary medicine and stated that 88% of its member states, corresponding to 170 member states, reported that their population uses traditional medicines to treat illnesses [8]. The wide diverse distribution of *Veronica* plants, from aquatic to dry steppe habitats and from sea-level to high alpine regions [9], could be related to the wide range of traditional uses within these cultures (Table 1). As an example, *Veronica peregrina* L. is useful for treating hemorrhage, gastric ulcer, infections, and diseases related to macrophage-mediated inflammatory disorders, as illustrated in Korean traditional medicine [10]. Tea made from *Veronica spicata* L. is a well-known remedy in traditional medicine [11]. *Veronica* species have cytotoxic and anti-inflammatory activity. *Veronica officinalis* L. (common speedwell) is used for treating liver, eczema, ulceration, snake bites, wound healing, and skin lesions in Balkan traditional medicine [12]. *Veronica* species are used in traditional medicine for the treatment of rheumatism [13], hemoptysis, laryngopharyngitis, hernia [14], and lung and respiratory diseases (e.g., against cough or as an expectorant) [15]. They also have properties such as antiscorbutic and diuretic, as well as wound healing [16]. Three *Veronica* species, namely, *V. officinalis*, *Veronica chamaedrys* L., and *Veronica herba* DAC, are used in traditional Austrian herbal drugs [17]. *V. officinalis* is a popular medicinal plant, used as a commercial herbal product in many European countries [18]. There are around 79 *Veronica* species in Turkish flora, 26 of which are endemic. Different parts of *Veronica* species are used as a diuretic, for wound healing, and against rheumatic pains in Turkish folk medicine [19].

Thus, based on this knowledge, *Veronica* plants are potential sources of nutraceuticals and functional ingredients with a wide spectrum of bioactivities. In fact, *Veronica* species represent a valuable source of biological active compounds. Among others, the extracts of *Veronica* plants show antioxidant, antimicrobial, antifungal, anti-inflammatory, scolicidal, and anti-cancer activities, as well as inhibitory potential on acetylcholinesterase, tyrosinase, lipoxygenase, and xanthine oxidase [20]. Overall, these species might be considered good candidates for industrial or pharmacological applications. Therefore, this review covers information about the phytochemical composition of *Veronica* plants, mainly focused on phenolics and iridoids. Their biological properties are also detailed, as well as recent food applications.

## 2. Phytochemical Characterization of *Veronica* Plants

**Figure 1:**
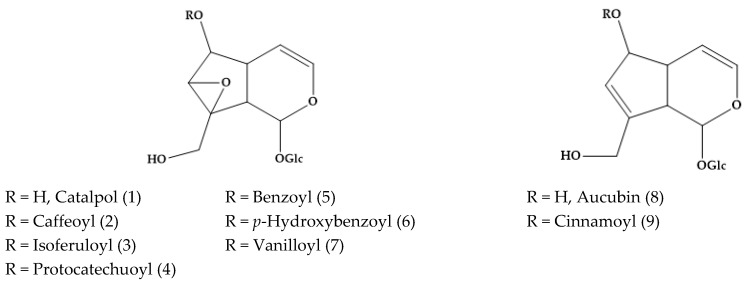
Catalpol and aucubin derivatives described in several *Veronica* plants, including sect. Paederota, Pseudolysimachia, Veronicastrum, Omphalospora, and Chamaedrys.

Several phytoconstituents isolated from the plant extracts were studied by means of mass spectrometry (MS) and spectroscopy techniques such as nuclear magnetic resonance (NMR). In the year 1973, Grayer-Barkmeijer and co-workers reported that catalpol (1) derivatives, i.e., caffeoyl-catalpol (2), isoferuloyl-catalpol (3), protocatechuoyl-catalpol (4), benzoyl-catalpol (5), *p*-hydroxybenzoyl catalpol (catalposide) (6), vanilloyl-catalpol (7), and cinnamoyl-aucubin (9), were isolated from several *Veronica* species, including sect. Paederota, Pseudolysimachia, Veronicastrum, Omphalospora, and Chamaedrys [21] (Figure 1). It depends on the species, e.g., neither of them was detected in *Veronica virginica* L. (sect. Paederota) and most of them were in *Veronica persica* Poir (Sect. Omphalospora). Verproside (6-*O*-protocatechuoylcatalpol) (4) was also isolated from *V. officinalis* [22]. In fact, Johansen and co-authors reported that 6-*O*-rhamnopyranosylcatalpol esters are chemical markers of *Veronica* sect. *Hebe* [23].

**Figure 2:**
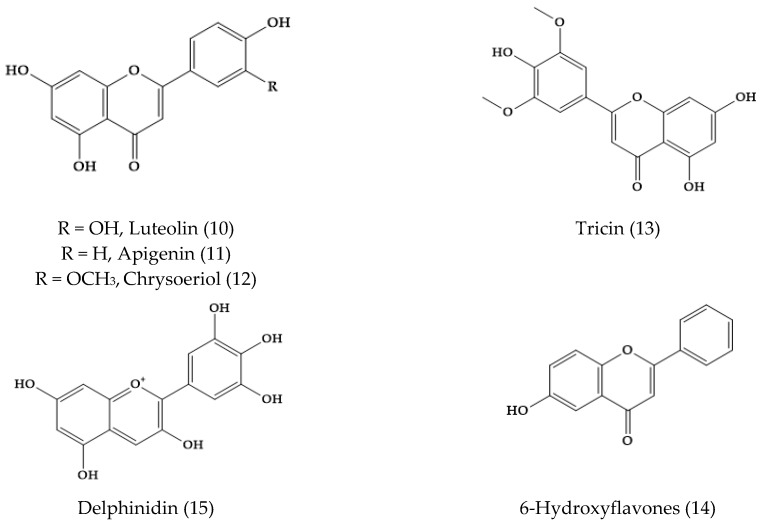
Common flavonoid aglycones in several *Veronica* species.

In *Veronica*, a larger variety of flavone aglycones was also found, e.g., luteolin (10), apigenin (11), chrysoeriol (12), tricin (13), and 6-hydroxyflavones (14) [24]. In fact, eight flavone aglycones were detected in 52 samples of 29 species of *Veronica,* and the most common ones were apigenin (11) and luteolin (10) (Figure 2) [25]. The observed exudate flavonoid aglycone profiles appeared to be characteristic for some related groups within *Veronica* genus, in consonance with the morphological, karyological, molecular, and other chemical data [25]. The presence of flavone glycosides was reported in several species, such as *Veronica gentianoides* Vahl., *Veronica alpine* L., and *Veronica fruticans* Jacq. The petals of the species of *Veronica,* like *Veronica gentianoides* Vahl, *Veronica arvensis* L., *V. persica, Veronica filiformis* Sm., *Veronica hederifolia* L., and *V. chamaedrys*, also showed the presence of the anthocyanidin delphinidin (15).

Furthermore, the structure of other particular compounds in *Veronica* species is detailed in this section; in addition to the expected iridoid glucosides, *Veronica* species are sources of new phytochemicals.

### 2.1. Veronica filiformis

**Figure 3:**
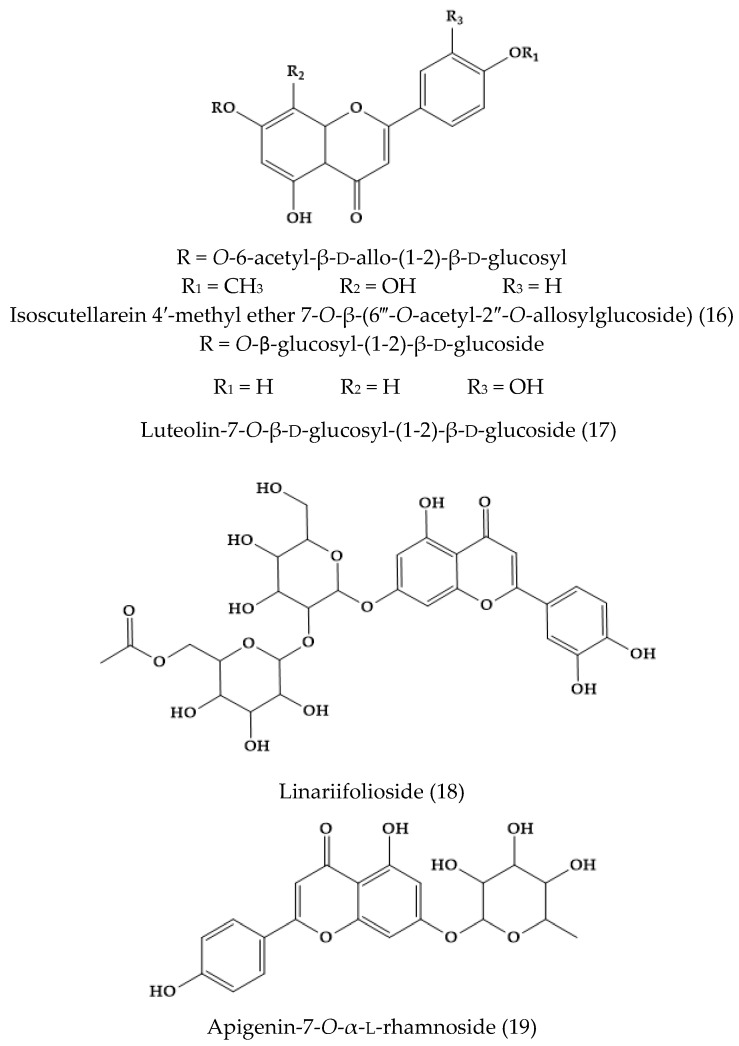
Flavone derivatives found in *Veronica filiformis* and *Veronica linariifolia*.

A non-common flavone glycoside was isolated from the whole plant of *V. filiformis* and identified by means of ^13^C NMR spectroscopy as isoscutellarein 4′-methyl ether 7-*O*-β-(6‴-*O*-acetyl-2″-*O*-allosylglucoside) (16) (Figure 3). This was the first report of 2-allosylglucose as a disaccharide unit of flavonoids [26].

### 2.2. Veronica linariifolia Pall. ex Link

**Figure 4:**
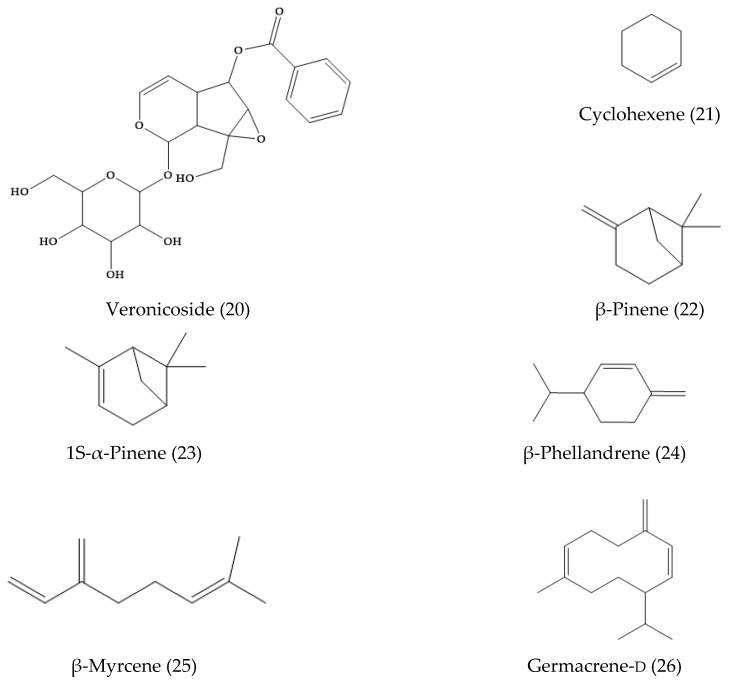
Phytochemicals from *Veronica linariifolia* essential oil.

A new flavonoid glycoside, linariifolioside (18) (Figure 3), was isolated from the alcohol extract of the dried whole herb. In addition, four known compounds, luteolin-7-*O*-β-d-glucosyl-(1-2)-β-d-glucoside (17), apigenin-7-*O*-α-l-rhamnoside (19), luteolin (10), and apigenin (11) were isolated from the same fraction and identified [27]. Another work showed the presence of 3′,4′,5,6,7-pentahydroxyflavone-7-*O*-β-d-glucosyl-(1″→2′)-β-d-glucoside, 4′,5,7-trihydroxy-3′,6-dimethoxyflavone-7-*O*-β-d-glucoside, apigenin-7-*O*-β-d-glucuronide methyl ester, apigenin-7-*O*-β-d-glucuronide ethyl ester, apigenin-7-*O*-β-d-glucuronide buthyl ester, apigenin (11), luteolin (10), vanillic acid, *p*-hydroxybenzoic acid, protocatechuic acid, protocatechuic acid ethyl ester, isoerulic acid, catechol, and emodin [28]. Alternatively, the essential oil was extracted by steam distillation approaches. The main compounds isolated were cyclohexene (21), β-pinene (22), 1S-α-pinene (23), β-phellandrene (24), β-myrcene (25), and germacrene-d (26) (Figure 4) [29].

### 2.3. Veronica fushii

**Figure 5:**
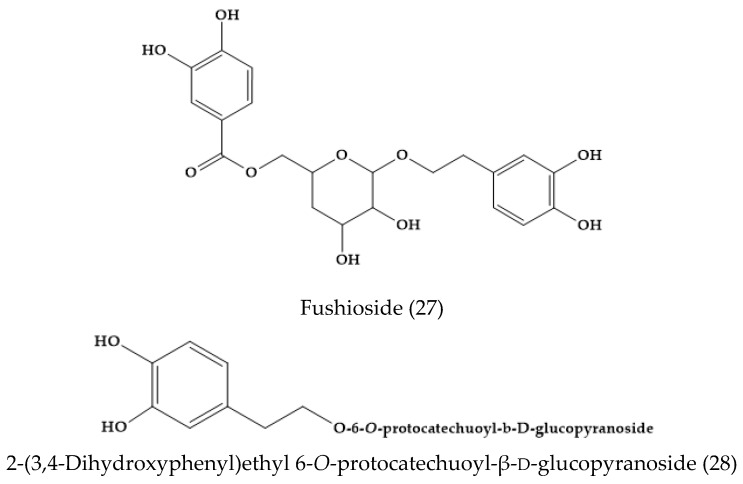

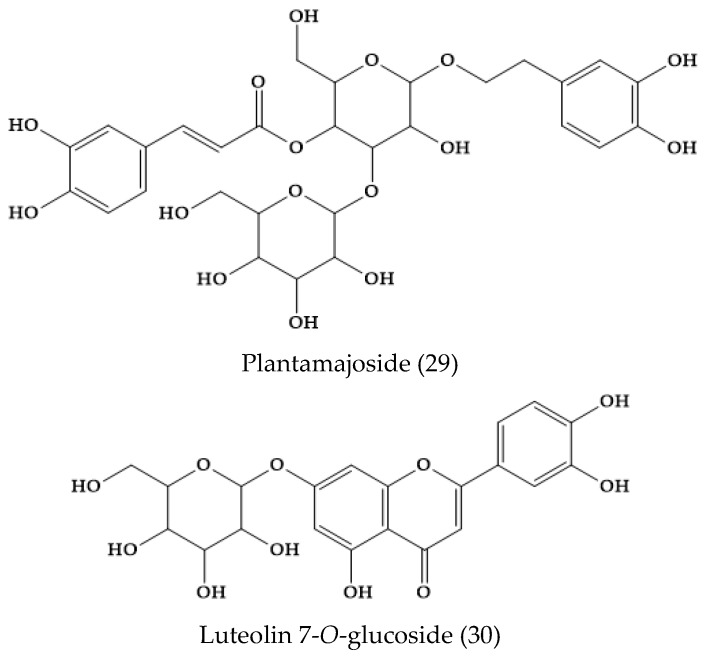
Phytoconstituents in *Veronica fushii*.

From the methanolic extract of the aerial parts of *V. fuhsii,* Ozipek and co-workers isolated and reported fushioside (27) and 2-(3,4-dihydroxyphenyl)ethyl 6-*O*-protocatechuoyl-β-d-glucopyranoside (28), along with a known phenylethanoid glycoside, plantamajoside (29), and a flavone glucoside, luteolin 7-*O*-glucoside (30) (Figure 5) [30]. Note that this species is endemic of Middle Anatolia [30], and it is not included in the plant list.

### 2.4. Veronica cymbalaria Bodard

**Figure 6:**
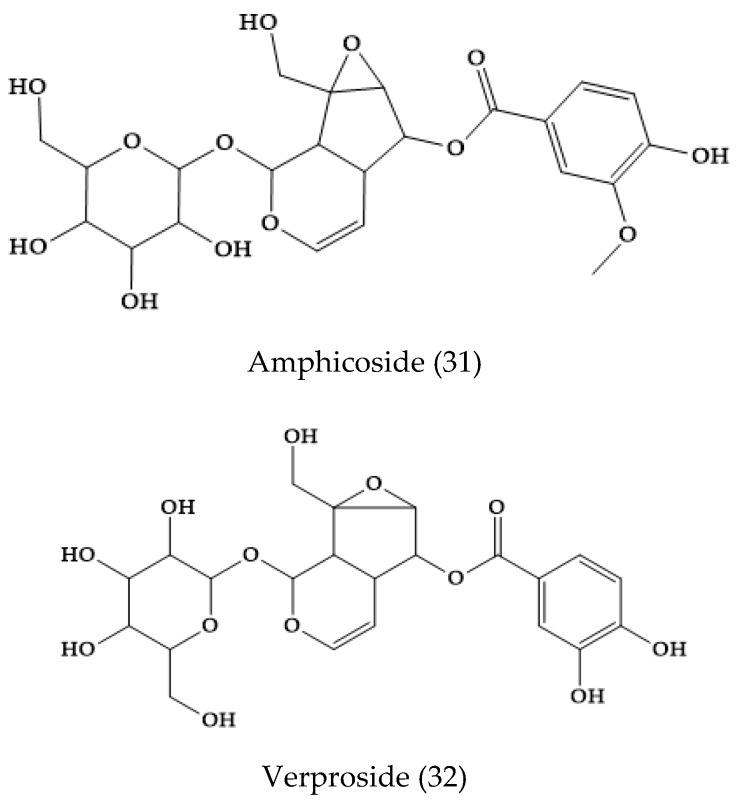

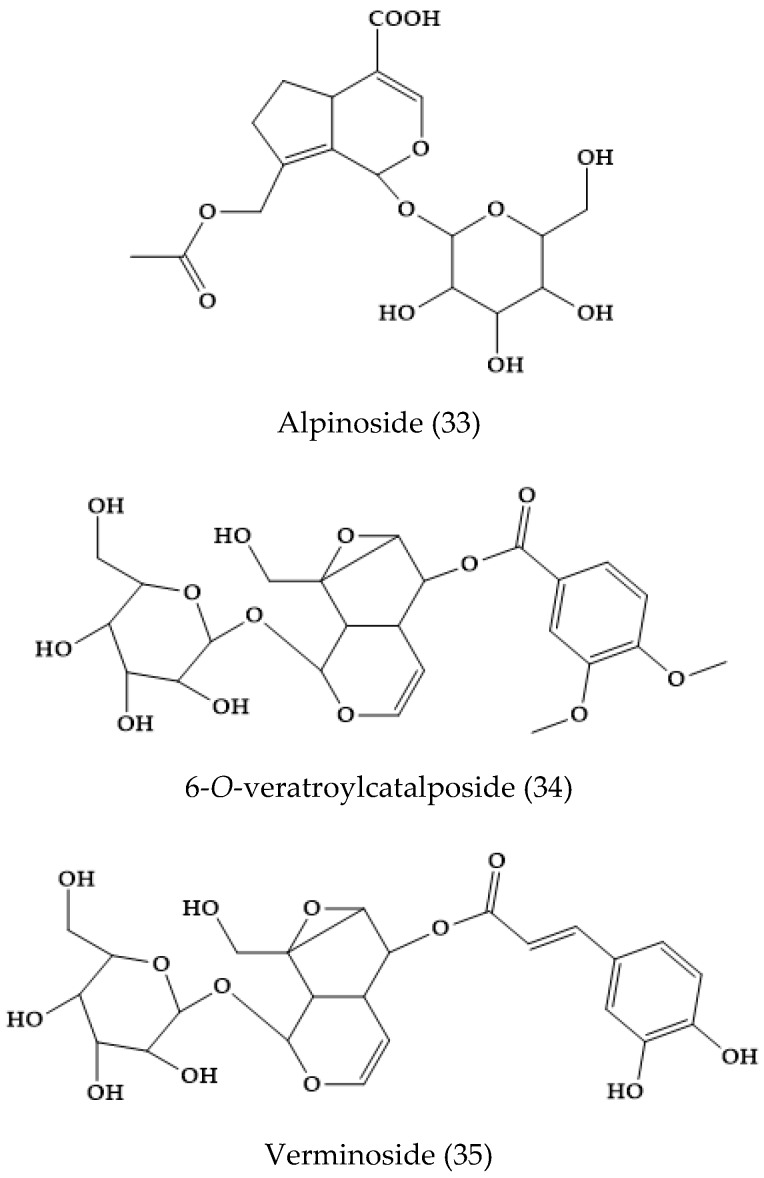
Phytoconstituents from *Veronica cymbalaria*.

The main iridoids and iridoid-phenolic constituents of extracts from this plant were catalpol (1), amphicoside (31), and verproside (32), together with alpinoside (33), aucubin (8), 6-*O*-veratroylcatalpol (34), and verminoside (35) (Figure 6). The iridoid alpinoside with a 8,9-double bond was found for the first time in genus *Veronica* [31]. In *V. cymbalaria,* other authors found the iridoid glucosides aucubin (8), catalpol (1), veronicoside (20), verproside (32), amphycoside, verminoside (35), catalposide (6), 6-*O*-veratroylcatalposide (34), and 6-*O*-isovanilloylcatalpol [32].

### 2.5. Veronica anagallis-aquatica L.

**Figure 7:**
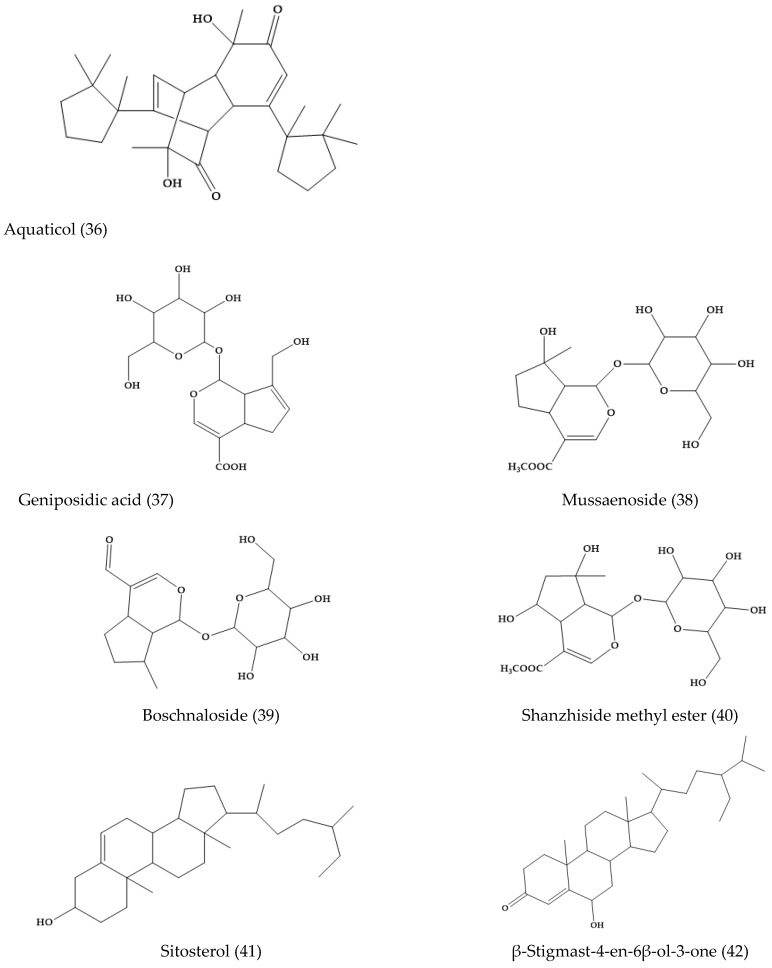
Selected phytochemicals from *Veronica anagallis-aquatica* and others.

Aquaticol (**36**), an unusual bis-sesquiterpene, was isolated from the methanol extracts of *V. anagallis-aquatica*. The other 11 well-known compounds are aucubin (**8**), geniposidic acid (**37**), mussaenoside (**38**), catalposide (**6**), verproside (**32**), amphicoside (**31**), catalpol (**1**), boschnaloside (**39**), shanzhiside methyl ester (**40**), sitosterol (**41**), and β-stigmast-4-en-6β-ol-3-one (**42**) (Figure 7) [33].

### 2.6. Veronica persica

**Figure 8:**
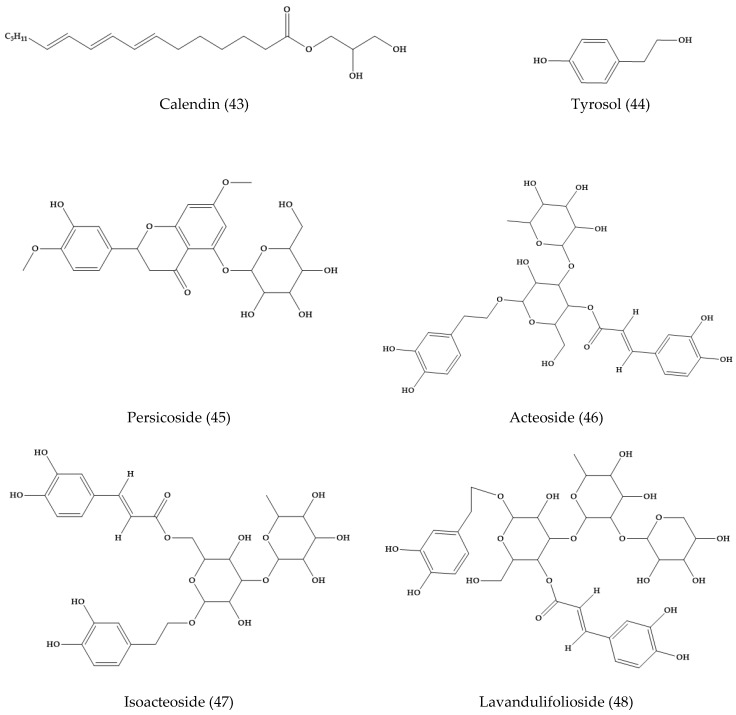
Phytoconstituents from *Veronica persica*.

*V. persica*, “common field speedwell” or “Persian speedwell”, is a neophytic weed originally from southwest Asia and widely distributed in the temperate regions. In dichloromethane extracts of this plant, calendin (**43**), tyrosol (**44**), and two benzoic acid derivatives were isolated [34] (Figure 8). Moreover, in the aerial parts of *V. persica*, a new phenylethanoid glycoside, persicoside (**45**), and three known phenylethanoid glycosides, acteoside (**46**), isoacteoside (**47**), and lavandulifolioside (**48**) (Figure 8), were isolated and the structure was confirmed by spectroscopic techniques.

In addition to phenylethanoid glycosides, hexitol, dulcitol, and seven known iridoid glucosides, aucubin (**8**), veronicoside (**20**), amphicoside (**31**), 6-*O*-veratroyl-catalpol (**34**), catalposide (**6**), verproside (**32**), and verminoside (**35**) were isolated from this plant species [35].

### 2.7. Veronica longifolia L. and Veronica liwanensis K. Koch

**Figure 9:**
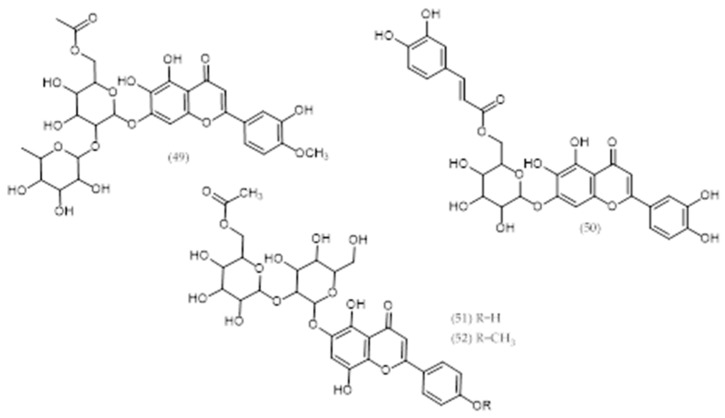
Phytoconstituents from *Veronica longifolia*, *Veronica liwanensis*, and *Veronica orientalis*.

Two new acylated 5,6,7,3′,4′-pentahydroxyflavone (6-hydroxyluteolin) glycosides and two unusual allose-containing acylated 5,7,8,4′-tetrahydroxyflavone (isoscutellarein) glycosides were isolated from *V. longifolia* and *V. liwanensis* and characterized by NMR spectroscopy as 6-hydroxyluteolin 4′-methyl ether 7-*O*-α-rhamnopyranosyl(1‴→2″)[6″-*O*-acetyl-β-glucopyranoside] (**49**) and 6-hydroxyluteolin 7-*O*-(6″-*O*-(*E*)-caffeoyl)-β–glucopyranoside (**50**), respectively (see Table 2 and Figure 9) [36]. Moreover, three chlorinated iridoid glucosides, asystasioside E (**92**) and its 6-*O*-esters, named longifoliosides A and B (**93**, **94**), were also detected [37].

### 2.8. Veronica orientalis Mill.

Isoscutellarein 7-*O*-(6‴-*O*-acetyl)-β-allopyranosyl(1‴→2″)-β-glucopyranoside (51) and its 4′-methyl ether (52) were obtained from *V. orientalis* species (Figure 9). The former was also found in *Veronica intercedens* Bornm. [36], although it is still an unresolved name [4].

### 2.9. Veronica thymoides P. H. Davis

**Figure 10:**
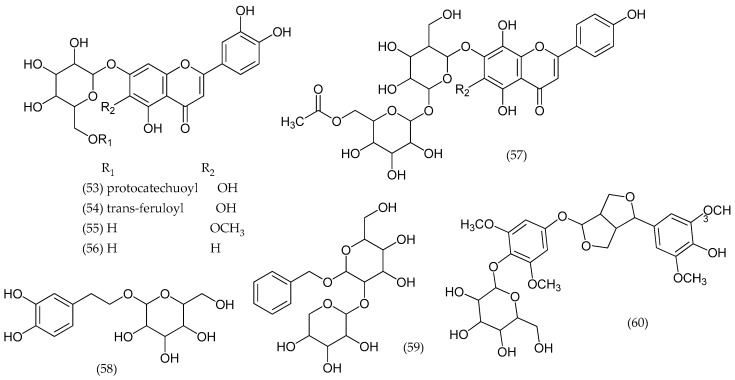
Phytoconstituents from *Veronica thymoides*.

From *V. thymoides* subsp. *pseudocinerea*, a new acylated flavone glucoside, 3′-hydroxyscutellarein 7-*O*-(6″-*O*-protocatechuoyl)-β-glucopyranoside (53), and a new phenol glucoside, 3,5-dihydroxyphenethyl alcohol 3-*O*-β-glucopyranoside were isolated, along with seven flavone, phenol, and lignan glycosides: 3′-hydroxyscutellarein-7-*O*-(6″-*O*-trans-feruloyl)-β-glucopyranoside (54), 3′-hydroxy, 6-*O*-methylscutellarein 7-*O*-β-glucopyranoside (55), luteolin 7-*O*-β-glucopyranoside (56), isoscutellarein 7-*O*-(6‴-*O*-acetyl)-β-allopyranosyl (1‴→2″)-β-glucopyranoside (57), 3,4-dihydroxyphenethyl alcohol 8-*O*-β-glucopyranoside (58), benzyl alcohol 7-*O*-β-xylopyranosyl (1″→2′)-β-glucopyranoside (59), and (+)-syringaresinol 4′-*O*-β-glucopyranoside (60) (Figure 10) [38].

### 2.10. Veronica arvensis

**Figure 11:**
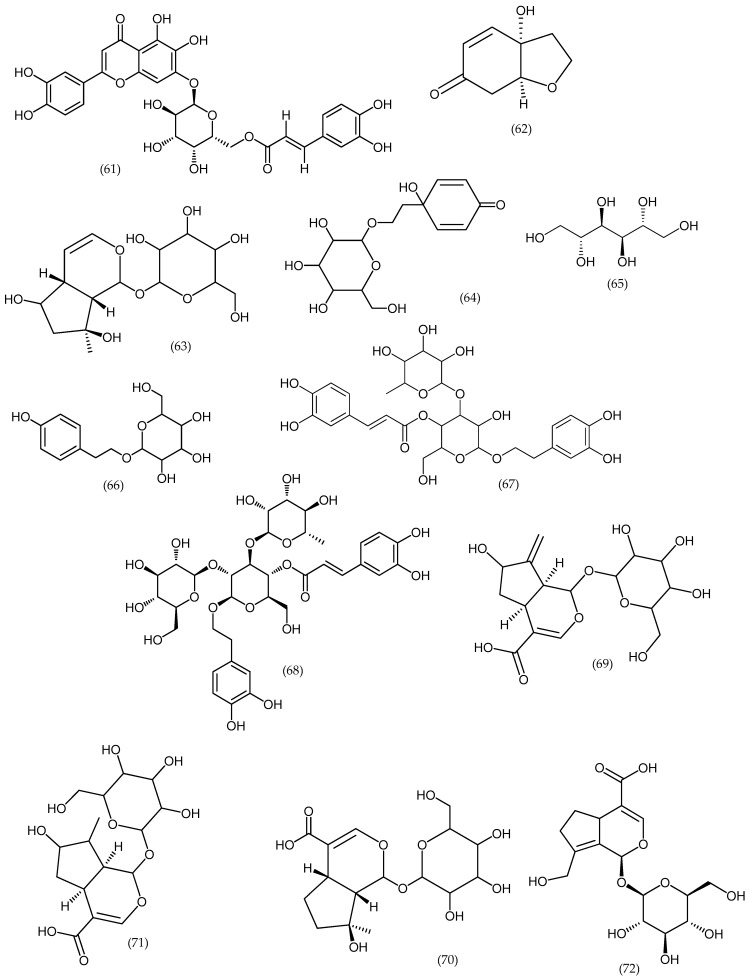
Phytoconstituents reported in *Veronica arvensis* and other species (Table 2).

The water extracts of this plant revealed the presence of cornoside aglycone and rengyolone (62). The iridoid glucoside, ajugol (63), and the phenylethanoid glucoside, cornoside (64) (Figure 11), were isolated from species of *Veronica* for the first time [39]. Other compounds are described in Table 2.

### 2.11. Veronica turrilliana Stoj. & Stef.

**Figure 12:**
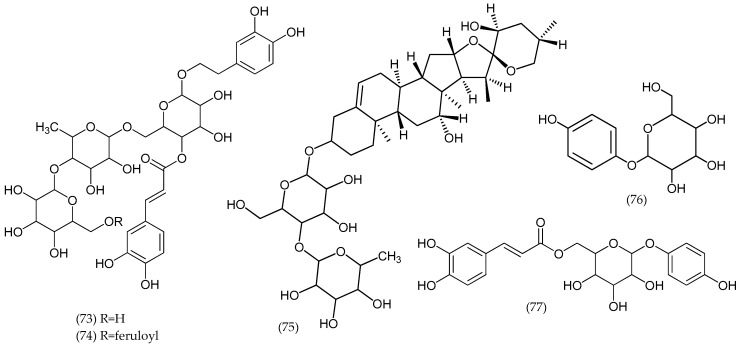
Phytoconstituents from *Veronica turrilliana*.

From *V. turrilliana* aerial parts, two phenylethanoid glycosides, turrilliosides A and B, and a steroidal saponin, turrillianoside, were isolated and their structures elucidated as β-(3,4-dihydroxyphenyl) ethyl-4-*O*-E-caffeoyl-*O*-[β-glucopyranosyl-(1→4)-α-rhamnopyranosyl -(1→6)]-β-glucopyranoside (73), β-(3,4-dihydroxyphenyl)ethyl-4-*O*-*E*-caffeoyl-[6-*O*-*E*-feruloyl -β-glucopyranosyl-(1→4)-α-rhamnopyranosyl-(1→6)]-β-glucopyranoside (74), and (23S,25S)-12β,23- dihydroxyspirost-5-en-3β-yl *O*-α-rhamnopyranosyl-(1→4)-β-glucopyranoside (75) (Figure 12), respectively. Other glucosides were reported, namely, catalpol (1), catalposide (6), verproside (32), amphicoside (31), isovanilloylcatalpol, aucubin (8), arbutin (76), and 6-*O*-*E*-caffeoylarbutin (77) [40].

### 2.12. Veronica cuneifolia D. Don

Column chromatography of iridoid fractions of *V. cuneifoia* subsp. *cuneifolia* methanol extract resulted in the isolation of verproside (32), verminoside (35), amphicoside (31), veronicoside (20), catalposide (6), and catalpol (1). The comparison of the iridoid fractions of *V. cuneifolia* subsp. *cuneifolia* and *V. cymbalaria* using an HPLC diode array detector (DAD) system with 40% MeOH showed that iridoid fractions of *V. cymbalaria* contained veratroylcatalpol (34), isovanilloylcatalpol, and aucubin (8) in addition to the compounds found in iridoid fractions [19]. Additionally, seven iridoid glucosides, aucubin (8), catalpol (1), veronicoside (20), verproside (32), amphycoside, verminoside (28), and catalposide (6), were identified [32].

### 2.13. Veronica derwentiana Andrews and Veronica catarractae G. Forst.

**Figure 13:**
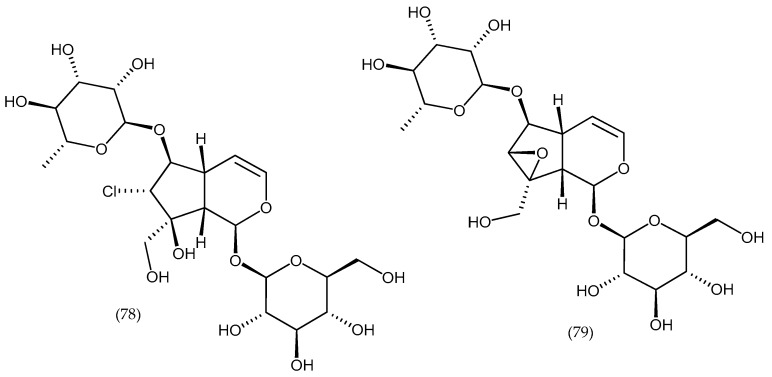
Structures of isolated compounds catarractoside and 6-*O*-rhamnopyranosylcatalpol (species *Veronica derwentiana* and *Veronica catarractae*).

Three unusual substituted benzoyl esters of aucubin were obtained from *V. derwentiana* and a chlorinated iridoid glycoside (catarractoside) (78) (Figure 13) from *V. catarractae*, in addition to other iridoids common to the genus. The chemical profile of *V. perfoliata* is similar to that of Northern Hemisphere species of *Veronica* because of the presence of characteristic 6-*O*-catalpol esters. The profile of *V. derwentiana* is unique, since 6-*O*-esters of aucubin rather than of catalpol dominate; however, the acyl groups are the same as those present in catalpol esters found in some other *Veronica* sections. *V. catarractae* also contains one of the catalpol esters characteristic of *Veronica,* in addition to three 6-*O*-rhamnopyranosyl substituted iridoid glycosides, one of which is 6-*O*-rhamnopyranosylcatalpol (79) (Figure 13) [41].

### 2.14. Veronica sibirica L.

**Figure 14:**
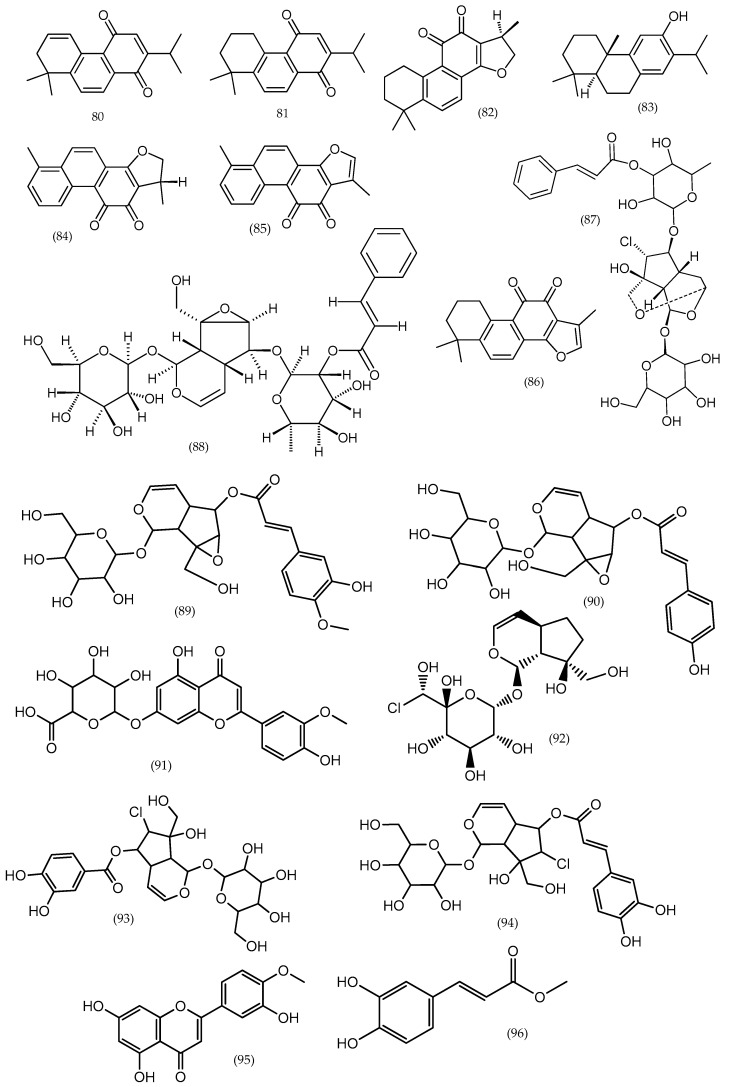
Several phytoconstituents in *Veronica sibirica* and *Veronica peregrina*.

Eight compounds were isolated from *V. sibirica* L. and identified as 1,2-dehydrocryptotanshinone, sibiriquinone A (80), sibiriquinone B (81), cryptotanshinone (82), ferruginol (83), dihydrotanshinone I (84), tanshinone I (85), and tanshinone IIA (86) [42]. A new iridoid glycoside, versibirioside (87), and a known iridoid glycoside, verbaspinoside (88) (Figure 14), were also reported from the whole plant. In addition, versibirioside was isolated from the whole plant of *V. sibirica* [43].

### 2.15. Veronica Peregrina L.

Eight iridoid glycosides and four phenolic compounds were isolated from the ethyl acetate extract of *V. peregrine*. The compounds were identified as protocatechuic acid, luteolin (10), veronicoside (20), minecoside (89), specioside (90), amphicoside (31), catalposide (6), 6-*O*-cis-*p*-coumaroyl catalpol, *p*-hydroxybenzoic acid methyl ester, verproside (32), verminoside (35), and chrysoeriol 7-glucuronide (91) by spectroscopic analysis (Figure 14) [12]. In the methanolic extract, the presence of chrysoeriol (12), diosmetin (95), 4-hydroxybenzoic acid, apigenin (11), caffeic acid methylester (96), and protocatechuic acid was reported [44]. The phenolic acid 4-hydroxybenzoic acid was also found by other authors [45].

### 2.16. Veronica montana L., Veronica polita Fr., and Veronica spuria L.

The phenolic compounds of *V. montana*, *V. polita*, and *V. spuria* showed that flavones were the major compounds (*V. montana*: seven phenolic acids, five flavones, four phenylethanoids, and one isoflavone; *V. polita*: 10 flavones, five phenolic acids, two phenylethanoids, one flavonol, and one isoflavone; *V. spuria*: 10 phenolic acids, five flavones, two flavonols, two phenylethanoids, and one isoflavone). *V. spuria* possessed the highest contents in all groups of phenolic compounds, except flavones [46].

### 2.17. Veronica spicata

**Figure 15:**
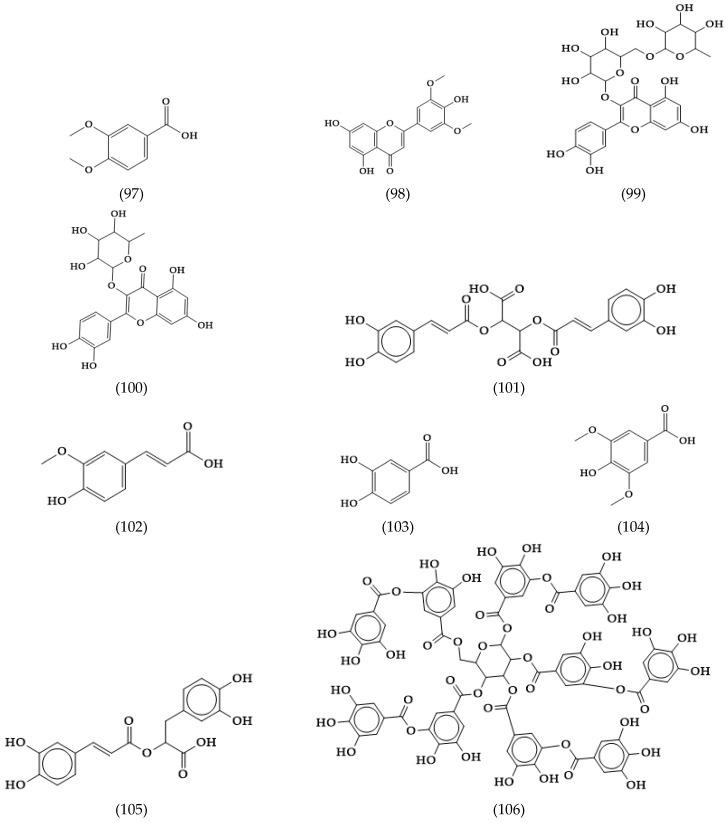
Other phytoconstituents in *Veronica spicata*.

Six 6-hydroxyluteolin glycosides acylated with phenolic acids were elucidated in this species, three of which were new compounds and called spicoside (61) derivatives. A flavonoid survey of seven more species belonging to subgenus *Pseudolysimachium* and eight species of *V*. subgenus *Pentasepalae* showed that all the *Pseudolysimachium* species and four of the *Pentasepalae* species produced 6-hydroxyflavone glycosides, whereas the remaining four *Pentasepalae* species contained acetylated 8-hydroxyflavone glycosides [39]. Moreover, from the species, 10 phenolic compounds were characterized: chrysin (98), rutin (99), and quercitrin (100), as well as cichoric (101), ferulic (102), protocatechuic (103), syringic (104), rosmarinic (105), and tannic acids (106) (Figure 15) [11].

### 2.18. Veronica officinalis

**Figure 16:**
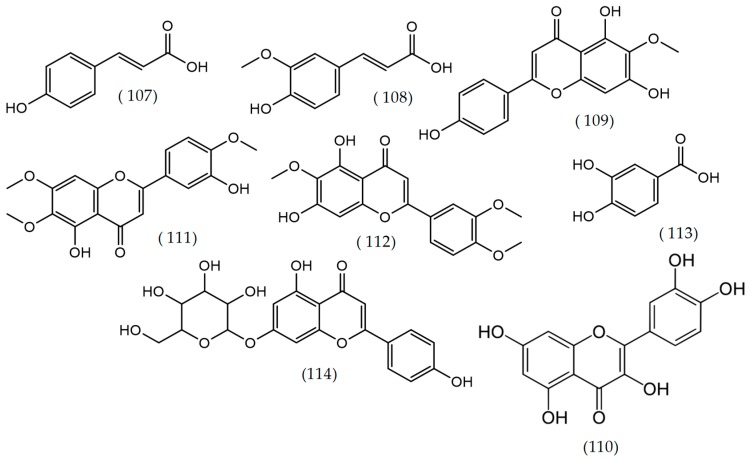
Structures of compounds reported in several *Veronica* species: *Veronica officinalis*, *Veronica ciliata*, and *Veronica rosea*.

The main phenolic compounds characterized in *V. officinalis* L. were *p*-coumaric acid (107), ferulic acid (108), luteolin (10), apigenin (11), quercitrin (100), hispidulin (109), quercetin (110) (Figure 16), and the sterol β-sitosterol (41). Some of these compounds (ferulic acid, coumaric acid, apigenin, and luteolin) were also found in *V. teucrium* L. and *V. orchidea* Crantz. Aglycones hispidulin, eupatorin (111), and eupatilin (112) (Figure 16) were also detected for the first time in the *Veronica* genus, mainly after hydrolysis, suggesting the presence of glycosylated forms. Eupatilin was found only in *V. orchidea* extracts. This chemical composition showed intravarietal differences [47].

### 2.19. Veronica ciliata Fisch.

Five main compounds, including two iridoid glycosides (catalposide (6), verproside (32)) and three phenolic compounds (luteolin (10), 4-hydroxy benzoic acid, and 3,4-dihydroxy benzoic acid (113)) (Figure 16), were isolated from the crude extract of *V. ciliata* by high-speed countercurrent chromatography [48].

### 2.20. Veronica rosea Desf.

The phytochemical study of butanolic extract of aerial parts of *V. rosea* led to the isolation and identification of four phytoconstituents: apigenin-7-*O*-β-glucopyranoside (114) (Figure 16), isoscutellarein-7-*O*-β-d-glucopyranoside, isoscutellarein7-*O*-[(6′’’-*O*-acetyl-β-d-allopyranosyl-(1→2)]-β-glucopyranoside, and mannitol (65) [49].

### 2.21. Others Phytochemicals and Species

**Figure 17:**
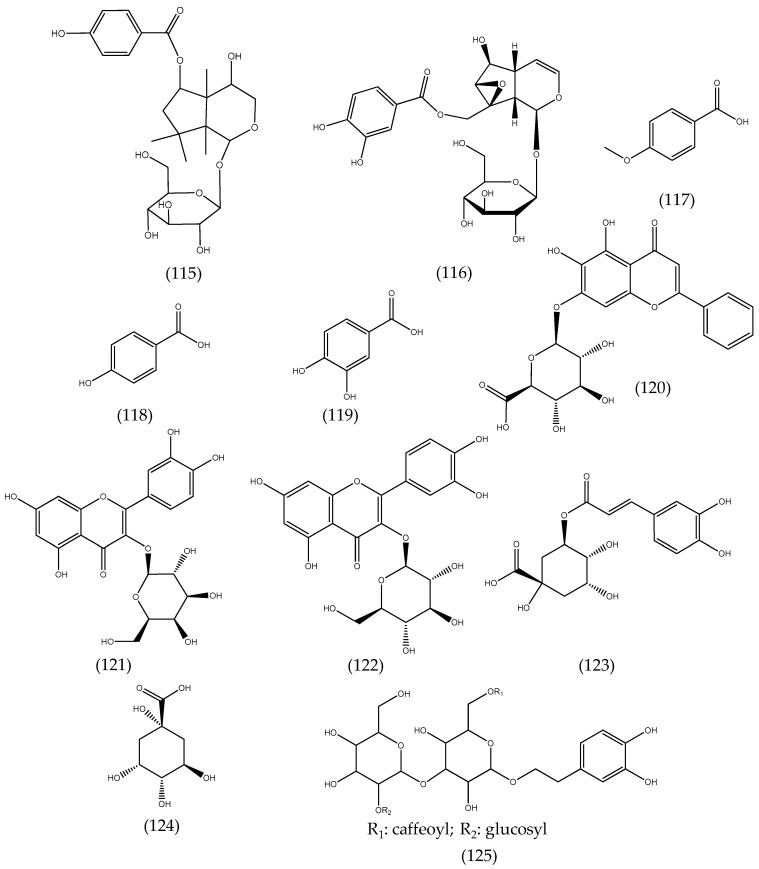
Structures of compounds reported in several Veronica species: *Veronica americana*, *Veronica jacquinii*, *Veronica teucrium*, and *Veronica urticifolia*.

*Veronica americana* Schwein. ex Benth. methanolic extracts revealed new iridoids identified as 4β-hydroxy-6-*O*-(*p*-hydroxybenzoyl)-tetrahydrolinaride (115) and 10-*O*-protocatechuyl-catalpol (116), along with four known aromatic compounds, veratric acid (97), *p*-methoxybenzoic acid (117), *p*-hydroxybenzoic acid (118), and protocatechuic acid (119) (Figure 17) [50]. Kroll-Møller and co-workers isolated iridoid glucosides from *Veronica hookeri* (Buchanan) Garn.-Jones and *Veronica pinguifolia* Hook. f. from New Zealand [51]. Thirty-three water-soluble compounds were isolated from *Veronica pulvinaris* (Hook. f.) Cheeseman and *Veronica thomsonii* (Buchanan) Cheeseman. Most of the isolated compounds were esters of phenylethanoid and iridoid glycosides [52]. A chemosystematic investigation of the water-soluble compounds in *Veronica cheesemanii* and *Veronica hookeriana* Walp. showed that both species contained mannitol (65), in considerable amounts, and some iridoids such as aucubin (8), catalpol (1), and their esters [53].

**Figure 18:**
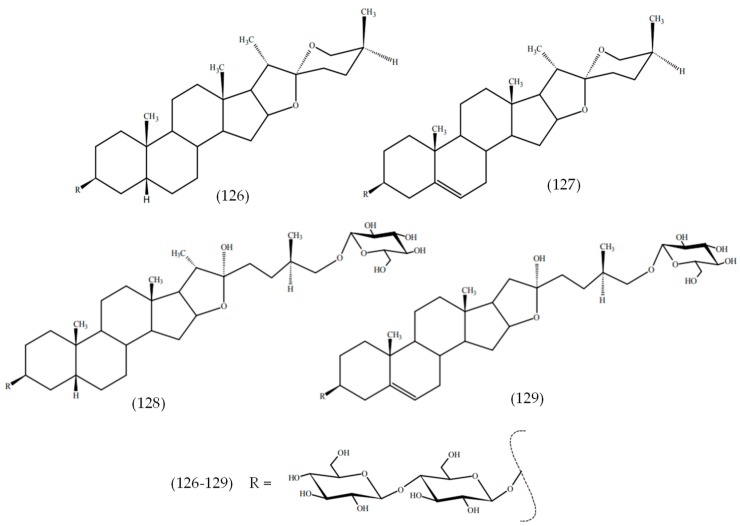
Structures of compounds reported in *Veronica beccabunga* L.

The phenolic compounds baicalin (120), hyperoside (121), isoquercetin (122), and chlorogenic acid (123), as well as quinic acid (124), were the main components in aqueous-acetone extracts of *Veronica jacquinii* Baumg., *Veronica teucrium* L., and *Veronica urticifolia* Jacq. (Figure 17) [13]. A new phenylethanoid triglycoside, chionoside J (125), was isolated from *Veronica beccabunga* L. (brooklime) [54], while two new spirostane glycosides, chamaedrosides C (126) and C1 (127), two new furostane glycosides, chamaedrosides E (128) and E1 (129), and two new furospirostane glycosides were found in *V. chamaedrys* (Figure 18) [55]. Compounds in other species are detailed in Table 2, i.e., *Veronica argute-serrata*, *Veronica biloba* schreb. ex L., *Veronica campylopoda* Boiss., *Veronica chamaedryoides* Engl., *Veronica dillenii* Crantz, *Veronica magna* M.A.Fisch., *Veronica micans* (M.A.Fisch.) Landolt, *Veronica micrantha* Hoffmanns. & Link, *Veronica orbelica*, and *Veronica vindobonensis* (M.A.Fisch.) M.A.Fisch.

## 3. Antimicrobial Activities of *Veronica* Plants

Many plants were used since ancient times to treat infections caused by bacteria that are now resistant to antibiotics [56]. There are several traditional uses of the genus *Veronica* that could be related to antimicrobial properties. For example, these species are used as expectorants, restoratives, tonics, and for the treatment of influenza and other respiratory diseases in traditional Chinese medicine [57]. In Romanian medicine, aerial parts of *Veronica* plants are known for their wound-healing properties and are also used for the treatment of cough and catarrh [47]. Despite their widespread usage in folk medicine, there is a lack of information about the antimicrobial activity. Only a few studies confirmed that certain *Veronica* species showed noticeable bioactivity such as antibacterial, antifungal, and antiviral activity.

### 3.1. Antibacterial Activity

Although *Veronica* species are traditionally used for their antibacterial effect, there are only a few studies that showed its strong antibacterial effect. The antibacterial activity against Gram-positive and Gram-negative species depends on the extract type (solvent used, part extracted, species, etc.). As an example, the antimicrobial properties of aerial parts of *V. spicata* extracts with ethyl-acetate, methanol, or water using the diffusion method and microdilution method were examined. The bacterial strains used in study were found to be susceptible toward methanol and ethyl-acetate extracts, with minimum inhibitory concentration (MIC) values between 1.25 and 5 mg/mL using the microdilution method, while aqueous extracts were inactive. The extracts prepared from leaves showed larger zones of inhibition compared to flowers and steam extracts of *V. spicata*, indicating stronger antimicrobial activity [11,58]. In another work and using the microdilution method, Živković et al. (2014) investigated the antibacterial effect of the *V. urticifolia* methanol extract against the Gram-negative bacteria *Escherichia coli, Enterococcus faecalis,* and *Pseudomonas aeruginosa*, and Gram-positive bacteria *Staphylococcus aureus, L. monocytogenes*, and *Bacillus cereus* [59]. After the measurement of the minimum bactericidal concentration (MBC) and MIC values, it was found that the most sensitive germ was *Staphylococcus aureus*. The antistaphylococcal effect is due to a main phenolic compound acteoside, which could inhibit the incorporation of leucine and disturb protein synthesis. The same antistaphylococcal effects were found in other studies [20]. Other chemical compounds could be also responsible for this antibacterial activity, e.g., β-sitosterol, campesterol, stigmasterol, hispidulin, and flavonoids. These results are important for human health because *S. aureus* is a pathogen germ difficult to treat with the development of antibiotic resistance. For this reason, natural alternative therapies to solve this problem are useful. Other studies showed the antibacterial activity of methanol, ethanol, or aqueous extracts from *Veronica urticifolia* Jacq., *Veronica orchidea* Crantz, *V. persica,* and *Veronica montana* L. against Gram-positive and Gram-negative bacteria [20,47,57,59]. The summary of the antimicrobial activity of different *Veronica* species is represented in Table 3.

As commented before, not all results were positive. Dulger and Ugurlu (2005) evaluated the antimicrobial activity of the methanol extracts obtained from endemic Plantaginaceae members from Turkey, including *Veronica lycica* E. Lehm. The antimicrobial activity was determined in *E. coli* (American Type Culture Collection (ATCC) 11230), *S. aureus* (ATCC 6538P), *Klebsiella pneumoniae* (UC57), *P. aeruginosa* (ATCC 27853), *Proteus vulgaris* (ATCC 8427), *B. cereus* (ATCC 7064), *Mycobacterium smegmatis* (Czech Collection of Microorganisms (CCM) 2067), *L. monocytogenes* (ATCC 15313), *Micrococcus luteus* (CCM 169), *Candida albicans* (ATCC 10231), *Rhodotorula rubra* (German Collection of Microorganisms (DSM) 70403), and *Kluyveromyces fragilis* (ATCC 8608) using the disc diffusion method. In this case, *V. lycica* had weak antimicrobial effect against the tested microorganisms [60]. *V. anagallis-aquatica* was tested using the agar well diffusion assay against five bacterial and two yeast strains. None of the extracts of this *Veronica* species showed significant inhibition comparing to the positive control (gentamicin) [61].

In some cases, the antimicrobial activity of the isolated compounds was determined. In a study conducted by Mocan and collaborators, *V. officinalis*, *V. teucrium*, and *V. orchidea* were selected from Romanian natural flora and investigated for their antioxidant and antimicrobial effects against anaerobic bacterial strains with emphasis on the isolated compounds, caffeic and chlorogenic acids. *V. teucrium* and *V. orchidea* presented a higher activity (MIC = 31.25 mg/mL and MBC = 62.5 mg/mL) than *V. officinalis* (MIC and MBC of 62.5 mg/mL), with the most sensitive strain being *Peptostreptococcus anaerobius*. All analyzed species contained both caffeic and chlorogenic acids, where the richest source of caffeic acid was *V. officinalis* and the highest amount of chlorogenic acid was found in *V. teucrium* [62].

### 3.2. Antifungal, Antiviral, and Antiparasitic Activity

Other studies demonstrated the antifungal effect of *Veronica* species [11,20,47]. Mocan and co-workers investigated the antifungal properties of *V. persica* against *Aspergillus niger* and *Penicillium hirsutum* using the Kirby–Bauer diffusimetric method. The antifungal effect was higher for *A. niger* than *P. hirsutum* and it is due to phenolic compounds [47]. Using the same method, antifungal effects of *V. persica* extract against *Candida albicans* and *A. niger* were demonstrated in another study [20]. The results showed that the highest antifungal effect for both fungal pathogens was obtained at a concentration 300 μg/mL of extract. Dunkic et al. demonstrated in another study the antifungal effect of methanol *V. spicata* extract at MIC values ranging from 1.25 mg/mL to 5 mg/mL. In addition, the methanol extract prepared only from *V. spicata*’s leaves also had an effect against the dermatophyte *Microsporum gypseum* [11]. As a result of these positive data, *Veronica* plants can be considered good natural therapeutic alternatives for mild fungal infections.

A new biological property, i.e., antiviral activity (against herpes simplex viruses HSV1 and HSV2), of *V. persica* was demonstrated in a recent research conducted by Sharifi-Rad et al. (2018) [63]. In this study, the ethanol extract of *V. persica* was tested on Vero cells infected with both types of viruses. The stronger antiviral activity was found in the 80% methanol fraction of *V. persica* extract during and after infection of the cells with viruses, thus suggesting the interference of the extract with the intracellular entry of the virus and a possible inhibition of the viral intracellular replication of endogenous herpetic viruses. HSV1 was much more sensitive to the action of the methanolic fraction compared to HSV2. In addition, the 80% MeOH fraction of *V. persica* extract administered to the cells at the same time with aciclovir (antiviral drug) showed a synergistic effect in reducing plaque formation by herpetic viruses [63]. This antiviral action indicates the usefulness of the *V. persica* extract in combination with the antiviral medication (such as aciclovir) for decreasing the severity of symptomatic episodes of oral herpetic infection, which relapses when the immune system is weak. Moreover, in a recent investigation conducted by these authors, in vitro and in vivo susceptibility of *Leishmania major* to *V. persica* extract was evaluated. Antileishmanial activity of plant extract was investigated on cultured *L. major* promastigotes and in mice. In vitro tests showed a high and dose-dependent inhibitory activity of plant extract, which was able to reduce the survival time of promastigotes in a concentration-dependent manner; for example, the survival time of promastigotes decreased to 10% at 750 μg/mL after 72 h of exposure time. There was a significant influence of *V. persica* extracts in vivo on accelerating the healing process, as well as reducing the overall disease burden, in an animal model by inducing nitric oxide production in macrophage cells [64].

## 4. Antioxidant Activities of *Veronica* Plants

### 4.1. In Vitro Studies

Medicinal plants, including *Veronica* species, are excellent sources of phytochemicals with potent antioxidant activities. Extracts from several species were tested in vitro through several methods such as DPPH (2,2-diphenyl-1-picrylhydrazyl) free-radical scavenging, the phosphomolybdate method [56,65,66,67,68], hydrogen peroxide scavenging and bleomycin-dependent deoxyribonucleic acid (DNA) damage test [69], oxygen radical absorbance capacity (ORAC) assay [12], ferric-reducing antioxidant power test [70], and ABTS (2,2-azinobis 3- ethylbenzothiazoline-6-sulfonate) radical scavenging ability [56].

Mocan and co-workers reported that ethanolic extracts of *V. officinalis*, *V. teucrium*, and *V. orchidea*, which contain phenolic acids and flavonoids, showed potent antioxidant activity [47]. The Trolox equivalent (TE) antioxidant capacity (TEAC) assay indicated that *V. officinalis* (157.99 ± 6.58 mg TE/g dry weight (d.w.)) and *V. orchidea* (155.41 ± 1.58 mg TE/g d.w.) exhibited similar antioxidant capacities, but higher than that of *V. teucrium* (96.67 ± 0.26 mg TE/g d.w.). This was also suggested using antioxidant activity measured with electron paramagnetic resonance (EPR) spectroscopy using Fremy’s salt. In another work, 14 *Veronica* species were tested for their radical scavenging activity against DPPH, superoxide (SO), and nitric oxide (NO) radicals. *V. chamaedrys* was the most active against SO radical (half maximal inhibitory concentration (IC_50_) 113.40 µg/mL) and *V. officinalis* against DPPH (IC_50_ 40.93 µg/mL) and NO radicals (IC_50_ 570.33 µg/mL) [68]. In another work, the antioxidant potential of various extracts obtained from aerial flowering parts of three *Veronica* species, *V. teucrium*, *V. jacquinii*, and *V. urticifola,* was evaluated in vitro by DPPH free-radical scavenging activity (IC_50_ values 12.58 to 66.34 µg/mL) and ferric-reducing antioxidant power assays (0.97 to 4.85 mmol Fe^2+^/g) [70]. *V. spicata* extracts obtained by different solvents (water, methanol, and ethyl-acetate) also demonstrated a radical scavenging effect [11], especially methanol ones. Although butylated hydroxytoluene and butylhydroxyanisole are generally more active [11,70], the former is a source of natural ingredients.

Antioxidant activities of *Veronica* species could be attributed to their content of iridoids and phenolic acids [65]. Moreover, acylated flavonoids and phenol glycosides from *V. thymoides* subsp. *pseudocinerea* exhibited potent radical scavenging activity against DPPH radicals [38]. The two phenylethanoid glycosides, turrilliosides A and B, isolated from *Veronica turrilliana* Stoj. & Stef. were found to be potent DPPH radical scavengers, approximately 1.6-fold better than the flavonoid quercetin [40]. In another work, the antioxidant capacity of *V. persica* phenolic-rich extracts was correlated with their total phenol content [56].

### 4.2. In Vivo Studies

The antioxidant activity of three *Veronica* species, *V. teucrium, V. jacquinii*, and *V. urticifola*, was examined in vivo in rats, and their effect on several hepatic antioxidant systems was tested, i.e., on the activity of glutathione peroxidase, glutathione reductase (GR), peroxidase (Px), catalase (CAT), and xanthine oxidase, as well as glutathione (GSH) content and level of thiobarbituric acid reactive substances (TBARS). Treatment with *Veronica* extracts (methanolic, aqueous-acetone, and water extracts) (100 mg/kg) inhibited CCl_4_-induced liver injury by decreasing TBARS level, increasing GSH content, and bringing the activities of antioxidative enzymes CAT, Px, and GR to control levels. The study suggested that the extracts analyzed could protect the liver cells from CCl_4_-induced liver damage by their antioxidative effect on hepatocytes [70]. *Veronica ciliata* Fisch. is also a considerable candidate for protecting liver injuries due to its antioxidant and anti-apoptosis properties [71].

The antioxidant activity of other *Veronica* extracts was associated with other bioactivities. As an example, Lu and co-authors reported that isolated compounds from *V. ciliata* showed anti-hepatocarcinoma activities against HepG2 liver hepatocellular carcinoma cells, which could be associated with its antioxidant activity [48]. Lee and co-workers evaluated the antioxidant activity, cytotoxicity, and collagen synthesis activity in vitro in order to test the anti-wrinkle effect of a formulated cream containing *V. officinalis* extract. Antioxidant evaluation was performed in fibroblast cells. The ethanolic extract showed good antioxidant activity against DPPH free radicals (103.50 µg/mL) and also exhibited a significant effect on collagen synthesis activity without cytotoxicity. In a placebo-controlled trial on women, the treatment with the formulated cream (Scoti-Speedwell) for 56 days significantly resulted in anti-wrinkle activity [72].

## 5. Anticancer Activities of *Veronica* Species

Plant-derived metabolites are beneficial sources of new anti-cancer drugs with reduced cytotoxicity and increased activity. More than 60% of today’s drugs with proven anticancer properties originated from plants [73]. Extracts obtained from the aerial parts of various *Veronica* species are globally used as folk medicine for the therapy of cancer [16]. Despite this fact, only a few amounts of *Veronica* species were investigated for their cytotoxic and anticancer activities (as an example, see Table 4). With the exception of one in vivo confirmation, all of the investigations were performed in vitro against various cancer cell lines. Also, there is a lack of mechanistic studies for compounds isolated from *Veronica* species.

### 5.1. In Vitro Studies

Methanolic and water extracts of several *Veronica* species were tested against cancer cells in vitro. Harput and collaborators studied the cytotoxic activity of five *Veronica* species: *V. cymbalaria*, *V. hederifolia*, *Veronica pectinata* L., *V. persica,* and *V. polita*. Their methanolic extracts showed dose-dependent cytotoxicity against KB (human epidermoid carcinoma) and B16 (mouse melanoma) cells. Additional fractionation of investigated extracts pointed out that the active compounds occurred in the chloroform fraction, but not the water one. Moreover, KB cells were more sensitive to the CHCl_3_-soluble parts of the extracts compared to B16 cells. Except for *V. cymbalaria*, *Veronica* species exhibited similar activity against KB cells, while *V. persica*, *V. polita*, and *V. pectinata* demonstrated potent activity against B16 cells [74]. Saracoglu et al. (2011) evaluated the cytotoxicity of aqueous extracts (10–800 µL) of *V. cuneifolia* subsp. *cuneifolia* and *V. cymbalaria* using the 3-(4,5-dimethylthiazol-2-yl)-2,5-diphenyltetrazolium bromide (MTT) assay against various cell lines, Hep-2 (human epidermoid carcinoma), RD (human rhabdomyosarcoma), and L-20B (transgenic murine L-cells), as well as in non-cancerous Vero cells (African green monkey kidney cells). Both samples showed cytotoxic activity against used cell lines, but *V. cuneifolia* subsp. *cuneifolia* showed a stronger effect with IC_50_ values ranging from 250.4 (for RD line) to 410.9 (for Vero cell line) µg/mL [75]. Moreover, a previous pharmacological investigation on edible *V. americana* (Raf.) Schwein species (American speedwell) showed that the methanolic extract of the aerial parts exhibited cytotoxic activity against colon (HF-6) and prostate (PC-3) human cancer cell lines with IC_50_ values of 0.169 and 1.460 µg/mL, respectively [76].

Other studies focused on the elucidation of the active compounds. As an example, the authors of Reference [50] conducted bioassay-guided fractionation of *V. americana* methanolic extract, employing the cytotoxic activity against two previously mentioned cancer cell lines in order to determine active components. Compounds in this extract that demonstrated activity higher than camptothecin (used as the positive control) were 4β-hydroxy-6-*O*-*(p*-hydroxybenzoyl)-tetrahydrolinaride and 10-*O*-protocatechuyl catalpol. Their activity was higher against HF-6 cells with IC_50_ values of 0.031 and 0.066 µM for americanoside and 10-*O*-protocatechuyl-catalpol, respectively. Also, according to the calculated selectivity index (SI), both compounds showed more selective cytotoxicity against applied cancer cell lines than against human normal MRC-5 (fetal lung fibroblast) cells. Yin and co-workers investigated anti-hepatocarcinoma activity of 95% ethanol extract of *V. ciliata* aerial parts and its fractions, using human hepatocellular carcinoma cells HepG2 and the 3-(4, 5-dimethylthiazol-2-yl)-2, 5-diphenyltetrazolium bromide (MTT) test. They demonstrated that active compounds were concentrated in the ethyl acetate fraction of the extract [77]. Iridoid compounds veronicoside, catalposide, amphicoside, and verminoside strongly inhibited HepG2 cell proliferation in a dose-dependent manner. Their inhibition rates (with the exception of veronicoside) were significantly higher compared to that of 5-fluorouracil used as a positive control, i.e., IC_50_ values ranged from 15.54 to 28.32 µg/mL, while the 5-fluorouracil IC_50_ value was 29.62 µg/mL. In the later study conducted by the same group of authors [48], the anti-hepatocarcinoma activities of five other compounds isolated from *V. ciliata* were also determined in vitro against the HepG2 cell line using the Cell Counting Kit-8 (CCK-8) method. The results showed that the proliferation of HepG2 cells was notably inhibited by these five compounds in a dose-dependent manner, and their activities decreased in the following order: luteolin > verproside > catalposide > 3,4-dihydroxy benzoic acid > 4-hydroxybenzoic acid, with IC_50_ values ranging from 102.36 to 444.76 µg/mL. The authors hypothesized that the exhibited activity of isolated compounds could be associated, at least in part, with their antioxidant activity. According to them, the higher cytotoxic potential of luteolin compared to analyzed iridoid glucosides and phenolic acids could be attributed to the higher number of phenolic groups in the molecule.

In a recent study, the cytotoxic properties of eight compounds isolated from *V. sibirica* were tested in vitro through the MTT assay against two cell lines: human neuroblastoma (SK-N-SH) and human hepatocellular carcinoma (BEL-7402). Compounds sibiriquinone A, cryptotanshinone, ferruginol, dihydrotanshinone I, tanshinone I, and tanshinone IIA showed beneficial inhibitory effects on the SK-N-SH cell growth, while compounds sibiriquinone A, cryptotanshinone, dihydrotanshinone I, and tanshinone IIA inhibited growth of the BEL-7402 cell line [42]. Moreover, the cytotoxic activity of iridoid compounds characteristic for *Veronica* species (aucubin, catalpol, and catalpol derivatives) was also determined against previously mentioned cell lines Hep-2, RD, and L-20B. Among tested compounds, verminoside showed very strong activity with IC_50_ values of 128 µM, 70 µM, and 103 µM, respectively. The activities of amphicoside and veronicoside were lower, while verminoside, verproside, and 6-*O*-veratroylcatalposide showed cytostatic activity [78]. Moreover, the in vitro antitumor activity of diterpenes, chemical compounds from *V. sibirica*, was also shown using the SK-N-SH human neuroblastoma cell line and BEL-7402 human hepatoma cell line [42].

### 5.2. In Vivo Studies

Tumors and different types of cancers are difficult to treat, but classical conventional chemotherapy can be combined with cytotoxic natural remedies. Živković and co-workers evaluated the antitumor activity of *V. urticifolia* methanolic extract in vivo in animals with Ehrlich ascites carcinoma (EAC) [59]. It is an aggressive, fast-growing carcinoma initially described as a spontaneous murine mammary adenocarcinoma [80]. The antitumor properties of *V. urticifolia* methanolic extract were estimated via determination of the tumor cell count, ascites volume, and cell viability, and the results were compared with those achieved for positive control *N*-acetyl-l-cysteine. Pretreatment (2 mg/kg body weight) for seven days before EAC implantation showed a statistically significant decrease in tumor cell viability, while ascites volume and tumor cell count were reduced to some extent, but not statistically significant.

There are too few data about the in vivo antitumor effects of compounds isolated from *Veronica* species. Acteoside (phenylpropanoid compound dominant in *V. urticifolia* extract) applied intraperitoneally (i.p.) in a concentration of 50 mg/kg in C57BL/6 mice as pretreatment for 13 days before implantation of melanoma cells induced a statistically significant increase in survival rate in animals [81]. According to the aforementioned in vitro studies, other candidates for the development of effective anticancer therapeutic agents could be verminoside, 4β-hydroxy-6-*O*-(*p*-hydroxybenzoyl)-tetrahydrolinaride, and 10-*O*-protocatechuyl catalpol.

## 6. Anti-Inflammatory Activity

### 6.1. In Vitro Studies

The anti-inflammatory properties of several *Veronica* species were evaluated in vitro and in vivo (Table 4). In particular, the anti-inflammatory effect of *Veronica peregrina* L. was demonstrated in a recent study [10]. Different concentrations of *V. peregrina* methanolic extracts were incubated with C57BL/6 mouse peritoneal macrophages and the release of pro-inflammatory mediators, cyclooxygenase-2 (COX_2_) and nitric oxide (NO), mediated by lipopolysaccharides was assessed; these were reduced dependent on the concentration of the extract. The mechanism of anti-inflammatory action was also highlighted: the inhibition of inducible nitric oxide synthase (iNOS) enzyme and consecutive decrease of NO production [10]. This study revealed the usefulness of *V. peregrina* extract as synergistic natural therapy in inflammatory diseases mediated by activated mast cells, such as arthritis, obesity, and atherosclerosis [82]. A similar study conducted by Harput et al. demonstrated the same biological anti-inflammatory activity of a methanol extract of five *Veronica* species which inhibited NO release from activated macrophages [74].

In another study, aqueous-acetone extracts of *V. teucrium*, *V. jacquinii*, and *V. urticifola* were tested for this effect on calcium ionophore-stimulated platelets, which release pro-inflammatory enzymes 12-lipoxygenase (12-LOX) and cyclooxygenase-1 (COX-1) with tromboxane B_2_ (TXB_2_) and prostaglandin E2 (PGE_2_). COX_1_ and its metabolite PGE_2_ were inhibited by *V. urticifolia* extract, while the 12-LOX enzyme was blocked only by the *V. jacquinii* extract (IC_50_ = 1.072 mg/mL). This anti-inflammatory activity is correlated with the presence of the main chemical compounds genistein, baicalin, isoquercetin, and hyperoside, but the mechanism of action is not yet completely known [13]. The results of these studies are important for human health, because natural remedies can successfully complete anti-inflammatory medical treatments.

Extracts of *V. chamaedrys* and *V. officinalis* also revealed in vitro anti-inflammatory activity on peroxisome proliferator-activated receptors (PPARs) and on the pro-inflammatory mediators, interleukin-8 (IL-8) and E-selectin [17]. Among the active constituents, *V. officinalis* extract rich in iridoid glycosides (verproside and verminoside) inhibited pro-inflammatory mediators via the nuclear factor kappa B (NF-κB) signaling pathway in a human lung cell line [15].

In another study, the anti-inflammatory action of *V. officinalis* extract in allergic inflammatory lung diseases was evaluated using A549 human lung epithelial cells. The results showed that the *V. officinalis* extract (40 to 160 μg/mL) inhibited in a dose-dependent manner the release of the main pro-inflammatory mediators IL-6 and IL-8, prostaglandin E_2_, and eotaxin. The data of the study did not clarify the mode of action of *V. officinalis* extract, but it is supposed that there is a link between the most abundant and important active compounds, iridoid glycosides (verminoside and verproside), and the anti-inflammatory effect [15]. These positive molecular results are in contrast to other studies that showed that the oral bioavailability of verproside is very low [83]. Nonetheless, more studies are required to elucidate the metabolites formed and their bioactivity. Moreover, an alternative way to administer *V. officinalis* extracts could be the pulmonary route using a spray for inhalation with the direct release of active substances to the lung epithelium [83].

### 6.2. In Vivo Studies

Inflammatory bowel diseases (IBD) (Crohn′s disease and ulcerative colitis) are a heterogeneous group of diseases with incomplete elucidate pathogenesis defined by inflammation, persistence, or recurrence in the gastrointestinal tract, different in terms of extension of lesions, symptoms, prognosis, and treatment [84]. The triggers of these diseases are complex and mediated by cytokines (interleukins, IL) [85] and signal transducer activator of transcription 3 (STAT3) [86,87], pro-inflammatory enzymes such as cyclooxygenase-2 (COX_2_) [88], and non-specific pro-inflammatory mediators, e.g., thromboxanes, prostaglandins, leukotrienes, oxygen free radicals, and NO [89]. Understanding these mechanisms led to research of new therapies, including natural alternatives. Thus, Akanda and co-workers investigated the anti-inflammatory property of *V. polita* species on experimental colitis induced in mice using dextran sulfate sodium (DSS) [90]. Forty mice divided into four groups were included in the study: control, mice treated with DSS, mice treated with DSS plus *V. polita* extract (200 mg/kg), and the last group received DSS associated with dexamethasone. The results of the study were surprising because, for the mice treated with *V. polita* associated with dexamethasone compared to those treated with DSS alone, the following data were obtained: pro-inflammatory cytokines (IL-1β, IL-6, tumor necrosis factor alpha (TNF-α)) were not activated and NO production in the intestine was reduced. In addition, COX-2, NF-κB, and the Janus kinase 2 (JAK2)/STAT3 signaling pathway were blocked in mice co-treated with DSS and *V. polita*, similarly to the group of mice co-treated with DSS and dexamethasone [90]. This can be attributed to the high content of total phenols and flavonoids of this species [46].

Kupeli et al. conducted a study concerning the antinociceptive and anti-inflammatory effect of the methanol and aqueous extracts of the species *V. anagallis-aquatica,* using a phenyl-*p*-benzoquinone (PBQ) writhing test and carrageenan-induced hind paw edema model in mice. Only the methanolic extract (500 mg/kg) showed significant antinociceptive and anti-inflammatory effects, which could be related to the presence of iridoid glucosides, catalposide and verproside. In addition, no toxic and adverse irritant gastric effects were revealed [16].

A new pharmaceutical formulation consisting of a microemulsion of *V. persica* was tested for anti-inflammatory effects on rat paw edema induced by two different substances—kaolin, stimulating pro-inflammatory cytokines, and dextran, increasing the release of histamine and consequently inducing vascular permeability. Inflammation induced by dextran was totally reduced following the co-administration of *V. persica* microemulsion compared to the kaolin inflammation. This effect was correlated with its high content of polyphenols and iridoids [65].

## 7. Other Properties

The neuroprotective and angiogenic effects of the methanolic and aqueous-acetone extracts of three *Veronica* species, *V. teucrium*, *V. jacquinii*, and *V. urticifolia,* were determined on human SH-SY5Y cell line neuroblastoma. The neurotoxicity was induced on the cells by oxidative and nitrosative stress using H_2_O_2_ and nitroprusside sodium, respectively. The six extracts showed a moderate neuroprotective effect, and this effect was higher for the cytotoxicity induced by oxidative stress and using *V. urticifolia* extract. Moreover, these authors showed that *Veronica* extracts, especially the *V. jacquinni* methanol extract and *V. teucrium* aqueous-acetone extract, showed antiangiogenic properties by preventing the formation of tubular structures in a Matrigel assay. Although this effect was probably due to the content of acteoside and aucubine, the mechanisms of action are unclear [79].

## 8. Food Applications of *Veronica* Plants and Other Uses

The enzymatic activity in foodstuffs result in some chemical reactions leading to food spoilage, thus making foods inedible or decreasing their qualities [91,92]. In addition to being an important health issue, which may sometimes lead to death, it may also yield great economic losses [93]. The World Health Organization reported that unsafe food resulted in illnesses in approximately two billion people throughout the world, and some of the cases were actually fatal [94]. There are some other methods that could prevent food spoilage, such as thermal processing; however, this technique might also result in a decrease in the nutritional value and quality of the food while reducing vegetative microorganisms. Therefore, to prevent food-related diseases and even death, a preservative should be used and this preservative has to be appropriate for the health of consumers and should not yield toxic materials [95]. Food manufacturers also search for compounds that could meet the expectations of healthy food trends, and this is also an important issue since synthetic preservatives are known to yield unwanted health results [96]. *Veronica* species have widespread traditional use throughout the world and, in addition to this usage, stems and leaves of some of the species are consumed as food in certain regions. Moreover, according to the Food Additive Status List by the Food and Drug Administration (FDA), *Veronica* species are listed as substances in conjunction with flavors that can be used in alcoholic beverages only [97]. However, since some members of this genus are also consumed by humans and used in traditional medicine, we can consider that usage of this plant is relatively safe [57].

Thus, the use of *Veronica* species in food preservation could be plausible with no adverse effects since some of them were demonstrated to possess antimicrobial effects (see Section 4). Although not directed on their use in food matrices, these studies also proved that the species extracts possess antibacterial effects and, thus, this genus has a potential to be used in food preservation [11,47,98,99,100]. In this sense, the antimicrobial effect of *Veronica* plants extracts against foodborne pathogenic and food contaminant bacteria was demonstrated by several authors, but with modest activity. As an example, the results of the experiment conducted by Mocan et al. (2015) on the antibacterial activity of *V. officinalis*, *V. teucrium*, and *V. orchidea* ethanolic extracts, tested using the microdilution assay, revealed that the most sensitive bacterial strains to *V. officinalis* were *Listeria monocytogenes* (ATCC 19114) and *Listeria ivanovii* (ATCC 19119), with the same values of minimum inhibitory (MIC) and minimum bactericidal concentration (MBC) of 7.81 mg/mL. In the case of *V. teucrium* antibacterial activity, the strains of *Staphylococcus aureus* (ATCC 49444), *Bacillus cereus* (ATCC 11778), and *Enterococcus faecalis* (ATCC 29212) showed equal values for MIC and MBC (7.81 mg/mL), being the most sensitive. The ethanolic extract of *V. orchidea* inhibited *L. monocytogenes and L. ivanovii* with MIC = 3.9 mg/mL and MBC = 7.81 mg/mL. Regarding the *V. officinalis* extract, *Bacillus cereus*, *Pseudomonas aeruginosa* (ATCC 27853), and *Escherichia coli* (ATCC 25922) were the most resistant species with MIC and MBC values higher than 15.62 mg/mL. According to Mocan et al. (2015), the activity of these extracts against Gram-positive bacteria like *Listeria* species and *S. aureus* could be related to their high β-sitosterol, campesterol, and stigmasterol content. Phenolic constituents such as apigenin, luteolin, and their glycosides could also contribute [11,20,47]. Another work also suggested that *V. montana* L. water extract and its main phenolic compound, protocatechuic acid, showed the highest antibacterial effect against *S. aureus* (ATCC 6538) (MIC and MBC, 7.5 mg/mL) but a poor effect against *B. cereus* (human isolate) (MIC = 22.5 mg/mL; MBC 45 mg/mL) [57].

The incorporation of these species in a food system was investigated recently. In this sense, the water extract of *V. montana* exerted antimicrobial effects against six pathogenic bacteria, but it was more effective against *L. monocytogenes* (MIC, 7.5 mg/L; MBC, 15.0 mg/mL). Its major compound, protocatechuic acid (15.7 mg/g), also showed antibacterial properties (MIC, 0.75 mg/L; MBC, 1.0 mg/mL) and it was evaluated after its incorporation in cream cheese, using this bacterium as a cheese contaminant. This compound preserved cream cheese by inhibiting the growth of *L. monocytogenes* at room temperature and in the refrigerator after three days of inoculation, without compromising the sensory properties. The compound was shown to alter the permeability of the bacterial cytoplasmic membrane, and this makes this plant species and its major component promising antibacterial food preservatives [57].

The antioxidant properties (see Section 5) can be also exploited to preserve food quality and reduce rancidity and off-flavors, but these studies were not carried out on food systems. In this context, further studies are required to establish their real applicability as natural preservatives instead of synthetic ones, including dosages, advantages, and disadvantages.

In another context, some wild edible plants are attracting attention as novel food ingredients for gourmet in agro-tourism, for example, for salads and refreshing candy products. Ricola^®^ (Laufen, Switzerland) sweets are made with *V. officinalis* in conjunction with other medicinal plants, used to refresh the mouth and throat. Blanco-Salas et al. (2019) suggested that *V. anagallis-aquatica*, among other wild plants in “Sierra Grande de Hornachos” (Spain), already entered the high Spanish hotel industry and small selected market niches, due to its sensory and nutritional characteristics [101]. As commented before, this is a natural source of iridoids and other phytochemicals.

## 9. Conclusions

More than 100 phytochemicals were identified in *Veronica* plants, which mainly belong to iridoid glycosides and phenolic compounds, particularly flavones and terpenoids. *Veronica* plants are described in traditional medicine for the treatment of many diseases, especially related to inflammatory disorders. In addition, they represent importance in cosmetic and food industries. The review of the literature highlights that *Veronica* plants have good antioxidant, anti-inflammatory, antimicrobial, and anticancer abilities, which are mainly related to the presence of iridoid glucosides and phenolic constituents. The antioxidant properties of *Veronica* species were determined through different in vitro and in vivo studies. In addition, *Veronica* plants showed interesting antimicrobial effects, and most studies focused on its effects against both Gram-positive and Gram-negative bacteria. This also included foodborne pathogens, such as *L. monocytogenes*. The anti-inflammatory studies agreed with the use of *Veronica* remedies for anti-inflammatory medical treatments. Many authors also hypothesized that the cytotoxic activity of *Veronica* plants is associated with their antioxidant and anti-inflammatory effects. However, studies proving the therapeutic effects of the *Veronica* genus in humans are scarce, with the exception of *V. officinalis* as an anti-wrinkle agent. Only few studies were conducted in vivo, while the molecular mechanisms of the pharmacotherapeutic effects remain still unknown. When looking at the food applications of *Veronica* plants, promissory reports showed that its incorporation into several food matrices, such as dairy products, results in an improvement of the shelf-life, through exerting antimicrobial and antioxidant effects. Thus, these plants may be conceived as upcoming and effective natural food preservatives.

## Figures and Tables

**Table 1:** List of some common *Veronica* species, their edibility, and medicinal uses [6].

Latin Name	Common Name	Edibility	Medicinal Use
*Veronica agrestis* L.	Field speedwell, green field speedwell	Yes	Yes
*Veronica americana* Schwein. ex Benth.	American brooklime, American speedwell	Yes	Yes
*Veronica anagallis-aquatica* L.	Water speedwell	Yes	Yes
*Veronica arvensis* L.	Corn speedwell	No	Yes
*Veronica beccabunga* L.	Brooklime, European speedwell	Yes	Yes
*Veronica catenata* Pennell		Yes	No
*Veronica chamaedrys* L.	Germander speedwell	Yes	Yes
*Veronica hederifolia* L.	Ivy-leaf speedwell	No	Yes
*Veronica longifolia* L.	Garden speedwell, long-leaf speedwell	Yes	No
*Veronica officinalis* L.	Common speedwell	Yes	Yes
*Veronica peregrina* L.	Necklace weed, neckweed, hairy purslane speedwell	No	Yes
*Veronica polita* Fr.	Gray field speedwell	Yes	Yes
*Veronica scutellata* L.	Marsh speedwell, skullcap speedwell	Yes	No
*Veronica spuria* L.	Bastard speedwell	Yes	No
*Veronica undulata* Wall.	Undulate speedwell	Yes	Yes
*Veronica strum virginicum* (L.) Farw.	Beaumont’s root, Culver’s root, Bowman’s root, Culver’s root, Black root	No	Yes

**Table 2:** Phytoconstituents obtained from ethanol extracts of some *Veronica* species (based on Reference [3]).

Species	Extract	Compounds
*Veronica argute-serrata*	Ethanol	Mannitol (65), catalpol (1), aucubin (8), gardoside (69), ajugol (63), mussaenosidic acid (70), epiloganica acid (71), arborescosidic acid (72), verbascoside-like compounds, acetyl-flavone glycoside
*Veronica arvensis* L.	Ethanol	Mannitol (65), cornoside (64), ajugol (63), salidroside (66), verbascoside-like compounds
*Veronica biloba* schreb. ex L.	Ethanol	Catalpol (1), aucubin (8), ajugol (63), epiloganic acid (71), alpinoside (33)
*Veronica campylopoda* Boiss.	Ethanol	Mannitol (65), catalpol (1), aucubin (8), ajugol (63), verminoside (35), acetyl-flavone glycoside
*Veronica chamaedryoides* Engl.	Ethanol	Verbascoside-like compounds; some iridoid
*Veronica dillenii* Crantz	Ethanol	Verbascoside (67) and cornoside (64)
*Veronica longifolia* L.	Ethanol	Mannitol (65), catalpol (1), aucubin (8), verposide, catalposide (6), verminoside (35), catalpol ester, flavones
*Veronica magna* M.A.Fisch.	Ethanol	Verbascoside-like compounds
*Veronica micans* (M.A.Fisch.) Landolt	Ethanol	Verbascoside (67) and cornoside
*Veronica micrantha* Hoffmanns. & Link	Ethanol	Mannitol (65), aucubin (8), verpectoside B (68), triterpene glycosides
*Veronica orbelica*	Ethanol	Verbascoside-like compounds
*Veronica vindobonensis* (M.A.Fisch.) M.A.Fisch.	Ethanol	Verbascoside (67) and cornoside (64)

**Table 3:** Summary of the antimicrobial activity of different *Veronica* species. MIC—minimum inhibitory concentration; MBC—minimum bactericidal concentration.

Species	Plant Part	Extract	Effect	Reference
*Veronica spicata* L.	Flowers and stem	Methanol and ethyl-acetate extracts	MIC values were between 1.25 and 5 mg/mL against *Staphylococcus aureus, Microccocus flavus, Listeria monocytogenes, Enterobacter cloacae, Escherichia coli, Bacillus cereus*, and *Pseudomonas aeruginosa*	[11,58]
*Veronica urticifolia* Jacq.	The aerial parts	Methanol extract	The most sensitive germ was *Staphylococcus aureus* (MIC and MBC = 7.5 mg/mL)	[59]
*Veronica lycica* E. Lehm.	The aerial parts	Methanol extract	The antimicrobial activity was determined against *E. coli*, *S. aureus*, *Klebsiella pneumoniae*, *P. aeruginosa*, *Proteus vulgaris*, *B. cereus*, *Mycobacterium smegmatis*, *L. monocytogenes*, *Micrococcus luteus*, *Candida albicans*, *Rhodotorula rubra*, and *Kluyveromyces fragilis*. The weak antimicrobial effect was observed against the tested microorganisms	[60]
*Veronica anagallis-aquatica* L.	The aerial parts	Methanol extract	The extracts were tested against five bacterial and two yeast strains. They showed significant inhibition compared to the positive control (gentamicin)	[61]
*Veronica officinalis* L., *Veronica teucrium* L., *Veronica orchidea* Crantz	The aerial parts	70% ethanol extract	Two anaerobic bacterial strains were used: *Peptostreptococcus anaerobius* and *Fusobacterium nucleatum*. *V. teucrium* and *V. orchidea* presented a higher activity (MIC = 31.25 mg/mL and MBC = 62.5 mg/mL) than *V. officinalis* (MIC and MBC of 62.5 mg/mL), with the most sensitive strain being *Peptostreptococcus anaerobius*	[62]
*V. officinalis, V. teucrium, V. orchidea*	The aerial parts	70% ethanol extract	Eight bacterial strains were used: *Staphylococcus aureus, Bacillus cereus, Listeria monocytogenes, Listeria ivanovii, Pseudomonas aeruginosa, Enterococcus faecalis, Salmonella typhimurium*, and *Escherichia coli*. The most sensitive strains were *Staphylococcus aureus, Listeria monocytogenes*, and *Listeria ivanovii* with MIC values between 3.9 and 15.62 mg/mL	[47]
*Veronica persica* Poir	The aerial parts	70% methanol extract	*V. persica* extract demonstrated an antifungal effect against *Candida albicans* and *Aspergillus niger* at a concentration 300 μg/mL of extract	[20]

**Table 4:** Some bioactive effects of *Veronica* plants and potential active compounds.

Type of Studies		Primary Outcomes	Active Compounds	*Veronica* spp.	References
In vitro	Human neuroblastoma cell line SH-SY5Y	Neuroprotective against H_2_O_2_ induced cytotoxicity	Iridoid glucosides acteoside, and aucubin (only in *V. urticifolia*)	*Veronica urticifolia* Jacq. *Veronica teucrium* L. *Veronica jacquinii* Baumg.	[79]
	Human endothelial cells EA.hy 926	Angiogenic	Phenylpropanoids and flavonoids	*V. jacquinii* *V. teucrium* *V. urticifolia*	
	Human lung epithelial cells A549	Anti-inflammatory in lung diseases (anti-asthmatic)	Iridoid glycosides (verminoside, verproside)	*Veronicaofficinalis* L.	[15]
	Human cancer cell lines HF-6 (colon), PC-3 (prostate) human normal MRC-5 cells (fetal lung fibroblast)	Cytotoxic	Iridoids	*Veronica americana Schwein. ex Benth*.	[50]
	SK-N-SH human neuroblastoma cell line, BEL-7402 human hepatoma cell line	Cytotoxic	Diterpenes	*Veronica sibirica* L.	[42]
In vivo	Phenyl-*p*-benzoquinone writhing test and carrageenan induced hind paw edema model in mice	Antinociceptive and anti-inflammatory	Iridoid glucosides, catalposide and verproside	*Veronica anagallis-aquatica* L.	[16]
	Rats′ paw edema induced by dextran	Anti-inflammatory	Phenolic compounds and iridoids	*Veronica persica* Poir	[65]
Clinical	Study design: randomized, placebo controlled for 58 days	Anti-wrinkles, antiaging of skin	Verbascoside	*V. officinalis*	[72]
